# Population increase and changes in behavior and morphology in the Critically Endangered Redonda ground lizard (*Pholidoscelis atratus*) following the successful removal of alien rats and goats

**DOI:** 10.1111/1749-4877.12500

**Published:** 2020-12-03

**Authors:** Colin M. DONIHUE, Jennifer C. DALTRY, Shanna CHALLENGER, Anthony HERREL

**Affiliations:** ^1^ UMR 7179 C.N.R.S/M.N.H.N. Département Adaptations du Vivant Paris France; ^2^ Department of Biology Washington University in St. Louis St. Louis Missouri USA; ^3^ Fauna & Flora International David Attenborough Building Pembroke Street Cambridge UK; ^4^ Department of Environment St. John's Antigua; ^5^ Environmental Awareness Group St. John's Antigua

**Keywords:** Antigua and Barbuda, Caribbean, conservation, endangered species, invasive alien species, island biology, Lesser Antilles, pest removal

## Abstract

Redonda is a small volcanic Caribbean island that is home to at least 4 endemic lizard species, including the Critically Endangered ground lizard (*Pholidoscelis atratus*). Black rats (*Rattus rattus*) and domestic goats (*Capra hircus*) were introduced to the island at some time after its discovery by Europeans in the late 1500s. They had a devastating effect on the island, resulting in the loss of nearly all trees and most of the ground vegetation. Point count surveys of *P. atratus* in 2012 indicated low densities, and the invasive rats were observed hunting and preying on the lizards. Both populations of rats and goats were successfully removed in 2017 as part of an ecological restoration program, and native vegetation and invertebrate populations have increased rapidly since. Population surveys in 2017, 2018, and 2019 show the lizard population has increased by more than sixfold. In 2017, as rats and goats were being removed, we evaluated the morphology and escape behavior of this species and repeated these measurements 1 year later. We observed that *P. atratus* had become bolder, with a reduced flight distance. We also detected changes in limb morphology related to locomotion and suggest possible explanations that will need to be further investigated in the future. These results show how the removal of invasive species can rapidly affect lizard population recovery and behavior, potentially restoring island ecosystems to their pre‐human interference dynamics.

## Graphical abstract

*Pholidoscelis atratus*, the Redonda ground lizard, is endemic to the small island of Redonda. Following a successful removal of alien rat and goats on Redonda, *P. atratus* population numbers have significantly increased and the lizards have become bolder. We also found changes in limb morphology suggesting the potential for rapid adaptation to the recovering island ecosystem.

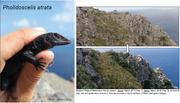

## INTRODUCTION

Invasive alien species are a global scourge, responsible for driving multiple species to extinction, imperiling fragile ecosystems, and causing billions of dollars in economic damage (Parmesan 2006; Simberloff *et al*. 2013). On islands especially, invasive alien species are rated as the number one cause of extinctions among endemic species (Russell *et al*. 2017; Spatz *et al*. 2017). While native island vertebrates represent only 5% of the Earth's terrestrial vertebrate diversity, they account for over 40% of the highly threatened species (Spatz *et al*. 2017). One of the most widespread and damaging groups of invasive alien species on islands are rats, which are responsible for numerous extinctions and an overall loss of biodiversity and ecosystem function (Pascal *et al*. 2004; Daltry 2006; Towns *et al*. 2006; Harper & Bunbury 2015; Spatz *et al*. 2017). Rats negatively impact island communities of birds (Duron *et al*. 2017), reptiles (Daltry 2007; Daltry *et al*. 2012), plants (Meyer & Butaud 2008; Pender *et al*. 2013) and invertebrates (St Clair 2011). Domestic goat is another pest that has been intentionally introduced to numerous islands. Goats have demonstrated a remarkable ability to survive on harsh and remote islands. Direct impacts of goats primarily involve the trampling and consumption of native vegetation, leading to a transformation in community structure (Chynoweth *et al*. 2013). Goats are therefore also considered major drivers of extinction (Coblentz 1978; Campbell & Donlan 2005) and have been labeled one of the most destructive herbivores introduced to island systems (Chynoweth *et al*. 2013). Consequently, many rat species and goats are increasingly the target of conservation efforts to remove these species from islands and prevent incursions (e.g. Russell *et al*. 2008, 2017). In addition to restoring and conserving the unique biodiversity of insular ecosystems, the removal of invasive species may provide a unique opportunity for experimental evolutionary studies investigating how insular populations evolve following major changes in their ecosystem.

**Figure 1:**
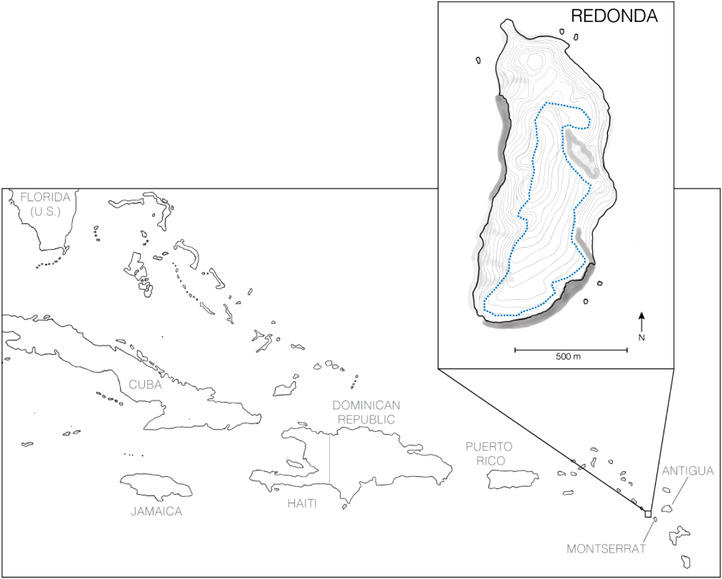
A map of Redonda, part of Antigua and Barbuda, in the Lesser Antilles. Blue dashed line reflects the transect used to survey *P. atratus* population in 2017 and 2018.

**Figure 2:**
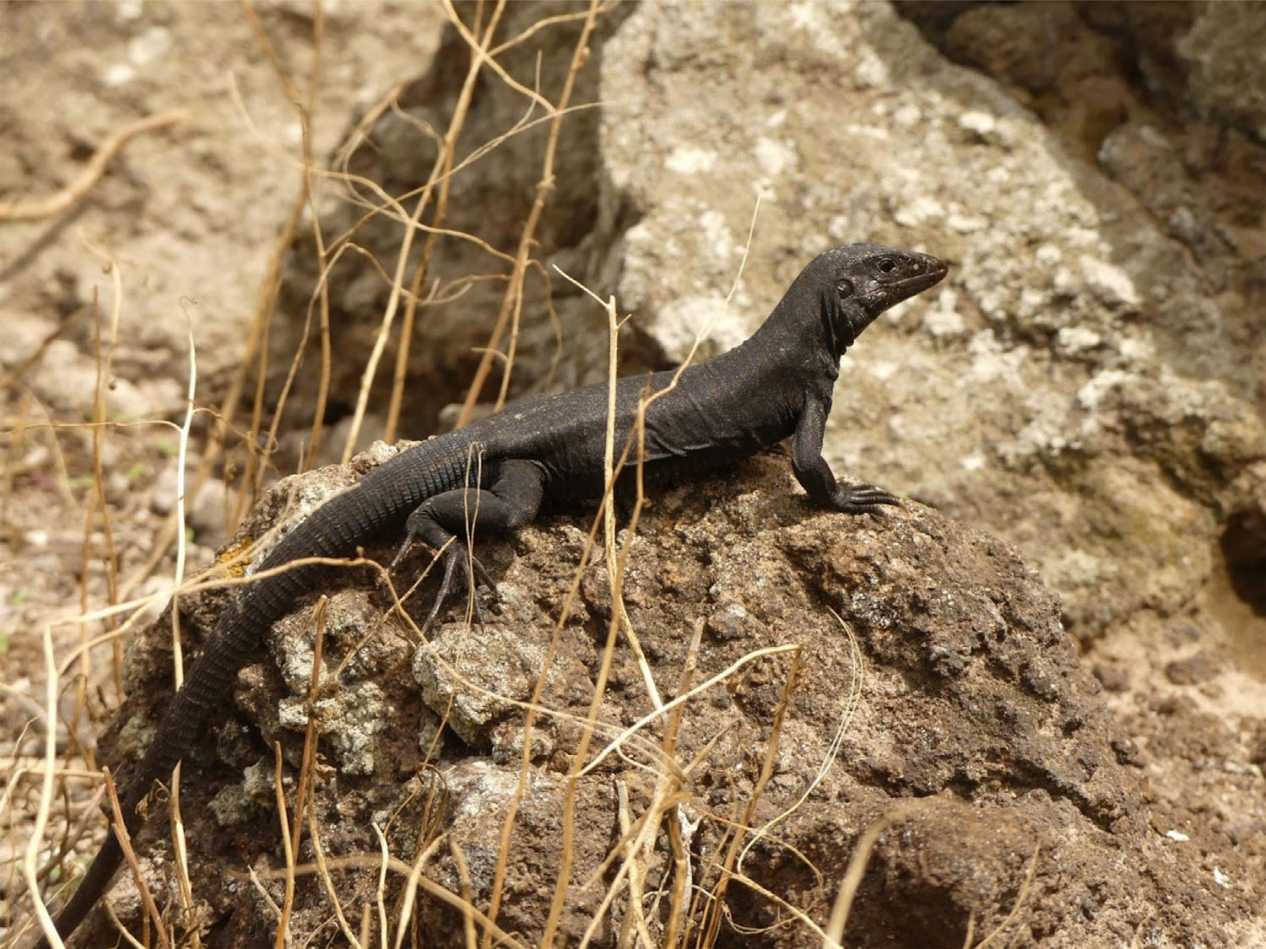
A photo of *P. atratus* on Redonda.

Here, we focus on the island of Redonda, a dependency of Antigua & Barbuda in the Lesser Antilles (Fig. 1). Redonda is surrounded on all sides by tall cliffs, ranging up to 400 m above sea level, and is separated by deep water from the islands of Nevis, Montserrat, and Antigua. Redonda harbors globally significant colonies of seabirds (such as brown, masked, and red‐footed boobies, magnificent frigatebirds, and red‐billed tropicbirds). In addition, thanks in part to its isolation, at least 4 endemic lizard species have evolved: the Critically Endangered Redonda ground lizard (*Pholidoscelis atratus*; Fig. 2; Goicoechea *et al*. 2016), the Redonda tree lizard (*Anolis nubilus*; Lazell 1972), the Redonda skink (*Copeoglossum redondae*; Hedges & Conn 2012), and an as‐yet unnamed dwarf gecko (*Sphaerodactylus* sp.; Daltry 2007). Dark‐colored iguanas (possibly *Iguana melanoderma*) were also historically recorded here (Bell & Daltry 2012; Breuil *et al*. 2020). The only native mammals on the island are bats.

Despite the imposing cliffs, humans introduced domestic goats (*Capra hircus*) after the island was first sighted on Christopher Columbus's 1493 voyage (the exact time of the goats’ arrival is unknown, but genetic studies show they are of Spanish origin: Amparo Martínez Martínez, Campus Universitario de Rabanales, *in litt*.). Black or ship rats (*Rattus rattus*) also colonized the island, having first reached the Caribbean as stowaways on ships from Europe. Redonda has largely remained uninhabited by humans except between 1860 and the 1920s, when the island was mined for seabird guano by the American Phosphate Company. Much like they have done elsewhere in the world, the rats (Pascal *et al*. 2004; Daltry 2006; Harper & Bunbury 2015) and goats (Coblentz 1978; Campbell & Donlan 2005; Chynoweth *et al*. 2013) contributed to the deforestation and desertification of Redonda, and are blamed for the extinction of the endemic skink (*Copeoglossum redondae*; Hedges & Conn 2012) and iguana, as well as the extirpation of the Antiguan burrowing owl (*Athene cunicularia amaura*; Bell & Daltry 2012) on the island. By 2012, the ecosystem was so severely degraded that even the c. 60 remaining feral goats were starving to death (Bell & Daltry 2012). The Government of Antigua and Barbuda, Environmental Awareness Group and Fauna & Flora International agreed to establish the Redonda Restoration Programme and remove the non‐native mammals with the goal of “*Significant recovery and regeneration of threatened species and habitats on Redonda”*. Operations to remove the goats ran from November 2016 through April 2017, and the rats were eradicated with brodifacoum from February to April 2017 (Lawrence *et al*. 2017; Daltry & Bell 2018).

To investigate the effect of invasive rat and goat removal on the native lizards, we collected data on the demography, morphology, and behavioral ecology of the Critically Endangered *Pholidoscelis atratus*. Ground lizards of the genus *Pholidoscelis* are active foragers that opportunistically feed on a variety of insects, plant material and even hermit crabs (Donihue *et al*. 2017). We estimated the effects of the removal of a predator (the rats) on the ground‐dwelling lizard's behavior by measuring flight initiation distance. This is a metric of how wary a lizard is, and it typically reflects predation pressure (Cooper & Whiting 2007; Brock *et al*. 2014; Cooper *et al*. 2014). Post‐eradication, we predicted a decrease in flight initiation distance. We further predicted that, post‐eradication, the abundance of lizards on the island would increase in response to a decrease in predation and improvement in habitat quality. Finally, we explored whether changes in predation pressure and vegetation regrowth had an impact on the morphology of the lizards, as previous studies have shown rapid changes in morphology following major alterations to the environment of lizards following translocation or catastrophic events (Herrel *et al*. 2008; Donihue *et al*. 2018).

## MATERIALS AND METHODS

As part of an initial feasibility study for removing invasive alien species, the population of the Redonda ground lizards was first surveyed in April 2012 using point counts (Bell & Daltry 2012). This rapid survey method is more commonly used for butterflies (e.g. Sparrow *et al*. 1994), birds (Bibby *et al*. 2000) and fish (Colvocoresses & Acosta 2007) but is suitable for active and conspicuous lizards at sufficiently high densities, including skinks (Havery *et al*. 2018) and ground lizards of the genus *Pholidoscelis* (Lawrence *et al*. 2013; Daltry & Bell 2018). On arrival at each point, the observer would sit or stand quietly for a 5‐min acclimatization period, and then silently count all lizards seen within a fixed radius (up to a maximum of 10 m, but reduced in vegetated areas with reduced visibility) for 10 min. Counts were conducted only during dry weather when the lizards were active. All points on Redonda were widely spread across the “safe zone” of the island that is accessible on foot (approximately 30 hectares). A total of 95 point counts were completed by JD and three colleagues in April 2012. This same methodology was repeated in March 2017 (at 65 points), March 2018 (25 points) and again in March 2019 (55 points). The number of counts varied depending on the availability of personnel. Based upon these counts and the areas of the point count circles, we extrapolated the mean number of individuals seen per hectare for each year. While we consider this a measure of relative density, this method has provided credible estimates of actual density for other species of *Pholidoscelis* when compared to mark‐recapture methods (e.g. Ward *et al*. 2010).

In addition, just as the rat eradication operation began in March 2017, additional population counts, behavior assays, and morphology data were collected for *P. atratus*. Exactly 1 year later, in March 2018, CMD and AH revisited the island and repeated those measurements to determine whether the lizard density, morphology, and behavior had changed following the eradication. We independently corroborated the density estimates obtained with point counts (above) using visual encounter surveys along a transect. In 2017, we defined a walking transect encircling the majority of the walkable surface of the island (Fig. 1). This transect was walked by the same observer (AH) on 2 afternoons in 2017 and 2 afternoons in 2018; along the transect, AH counted all *P. atratus* individuals that were sighted within approximately 3 m of the transect. To best mitigate known shortcomings of the visual encounter survey approach, all surveys were conducted by the same observer (AH), at the same time of day (1300–1500), and in favorable weather conditions (full sun to partly cloudy).

To assess the wariness of the lizards, we measured flight initiation distances of approximately 50 *P. atratus* individuals in both years. To do so, the same observer (AH) approached the animals at a consistent pace. We then recorded the distance between the observer and the lizard at the point of closest approach, the distance that the lizard fled, and the final distance the lizard settled post‐flight from the observer.

We measured morphology for 30 adult *P. atratus* individuals in 2017 and 33 adult individuals in 2018 using digital calipers (Mitutoyo; see Table 1). We captured both males and females and animals were sexes by inspecting the tail base for the presence of a hemipenis. We measured snout–vent length (mm), head dimensions (length, width, and depth of the head, lower jaw length, distance from quadrate to the tip of the lower jaw, distance from the back of the jugal to the tip of the lower jaw), body dimensions (body height, width, and inter‐limb distance) and limb dimensions (femur, tibia, metatarsus, longest toe on the hind limb, humerus, radius, metacarpus, and longest toe on the forelimb). Body mass was measured using a Pesola spring balance.

**Table 1:** Summary of the morphology and escape behavior metrics of *P. atratus* measured in 2017 and 2018

Morphology										Escape behavior				
	Snout‐to‐vent length					Humerus					Flight initiation distance			
	Males		Females			Males		Females			Males		Females	
2017	95.27 ± 2.2	19	76.90 ± 3.1	11	2017	14.40 ± 0.4	19	11.36 ± 0.4	11	2017	226 ± 17	23	176 ± 13	24
2018	110.63 ± 1.5	21	93.75 ± 2.1	12	2018	18.27 ± 0.2	21	14.89 ± 0.3	12	2018	168 ± 17	25	114 ± 10	25
	Head length					Femur					Total flight distance			
	Males		Females			Males		Females			Males		Females	
2017	23.33 ± 0.5	19	19.01 ± 0.7	11	2017	18.07 ± 0.5	19	14.22 ± 0.5	11	2017	75 ± 9	23	60 ± 9	24
2018	27.66 ± 0.4	21	22.77 ± 0.4	12	2018	22.01 ± 0.3	21	17.12 ± 0.4	12	2018	130 ± 20	25	102 ± 17	25
	Head width					Total forelimb					Final resting distance			
	Males		Females			Males		Females			Males		Females	
2017	11.77 ± 0.3	19	9.44 ± 0.3	11	2017	38.91 ± 1.0	19	30.99 ± 1.1	11	2017	276 ± 23	23	218 ± 12	24
2018	14.14 ± 0.2	21	11.19 ± 0.3	12	2018	48.33 ± 0.6	21	39.06 ± 0.7	12	2018	249 ± 26	25	177 ± 14	25
	Head height					Total hind limb								
	Males		Females			Males		Females						
2017	10.63 ± 0.3	19	8.76 ± 0.3	11	2017	72.78 ± 1.4	19	57.2 ± 2.1	11					
2018	13.19 ± 0.2	21	10.22 ± 0.2	12	2018	84.55 ± 1.0	21	68.11 ± 1.2	12					

## RESULTS

**Figure 3:**
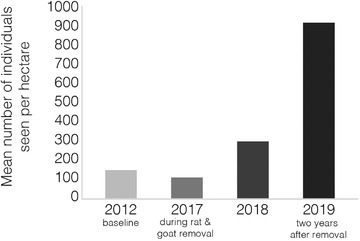
Mean number of *P. atratus* per hectare in 2012 (baseline survey), 2017 (during the removal of invasive rats and goats), 2018 (1 year after rats and goats were removed), and 2019 (2 years after)

The mean relative density of *P. atratus* on Redonda decreased between 2012 and 2017 from 146.9 to 111.7 individuals per hectare (Mann–Whitney *U* = 2565, *P* = 0.028). However, following the goat and rat removal, the relative density rose by almost threefold to 308.5 individuals per hectare in 2018 (a significant increase from 2017: *U* = 167, *P* < 0.0001) and tripled again to 935.3 individuals per hectare in 2019 (a significant increase from 2018: *U* = 216, *P* < 0.0001) (Fig. 3). We also counted, on average, more *P. atratus* along the transect in 2018 compared to 2017. In 2017, we saw an average of 91.5 lizards on the transect, and in 2018 that number had increased to 136. As lizards were not captured as part of the visual survey we did not differentiate between life stages.

In 2018, the *P. atratus* of both sexes were larger (SVL) than in 2017 (*t*_60_ = 7.50, *P* < 0.001; Table 1). Controlling for differences in body size, we found a significant difference in head shape in male *P. atratus*. Head shape was not significantly different between the sampling years for females (*P* > 0.05). Male heads were proportionately longer (*t*_58_ = 2.67, *P* = 0.010), wider (*t*_58_ = 3.66, *P* = 0.001), and taller (*t*_58_ = 3.26, *P* = 0.002) for a given snout–vent length. We also detected differences in the forelimbs of the lizards between the 2 sampling years. Humeri were longer for both sexes (*t*_59_ = 5.968, *P* < 0.001), as were radii (*t*_59_ = 3.85, *P* < 0.001). We detected no differences in the remaining forelimb dimensions (all *P* > 0.05). Males also had longer femora (*t*_58_ = 2.84, *P* = 0.006) and tibiae (*t*_59_ = 3.89, *P* < 0.001). The remaining hind limb dimensions were statistically indistinguishable between the years. This resulted in the total length of the forelimbs being significantly longer in 2018 compared to 2017 (*t*_58_ = 6.84, *P* < 0.001), when accounting for differences in body size with an SVL covariate. The difference in the sum length of the hind limb elements was only marginally different (*t*_59_ = 1.94, *P* = 0.057). The ratio of the length of the forelimbs to hindlimbs, when corrected for body size (SVL as co‐variate), was, however, larger in 2018 than in 2017 (*t*_58_ = 5.01, *P* < 0.001).

We found that flight initiation distance significantly decreased in 2018 (*t*_94_ = −3.78, *P* < 0.001). After being approached, *P. atratus* lizards in 2018 fled significantly further than the population had in 2017 (*t*_94_ = 4.08, *P* < 0.001), although the final distance from the observer post‐flight was significantly lower in 2018 (*t*_94_ = −2.31, *P* = 0.023; Table 1).

## DISCUSSION

**Figure 4:**
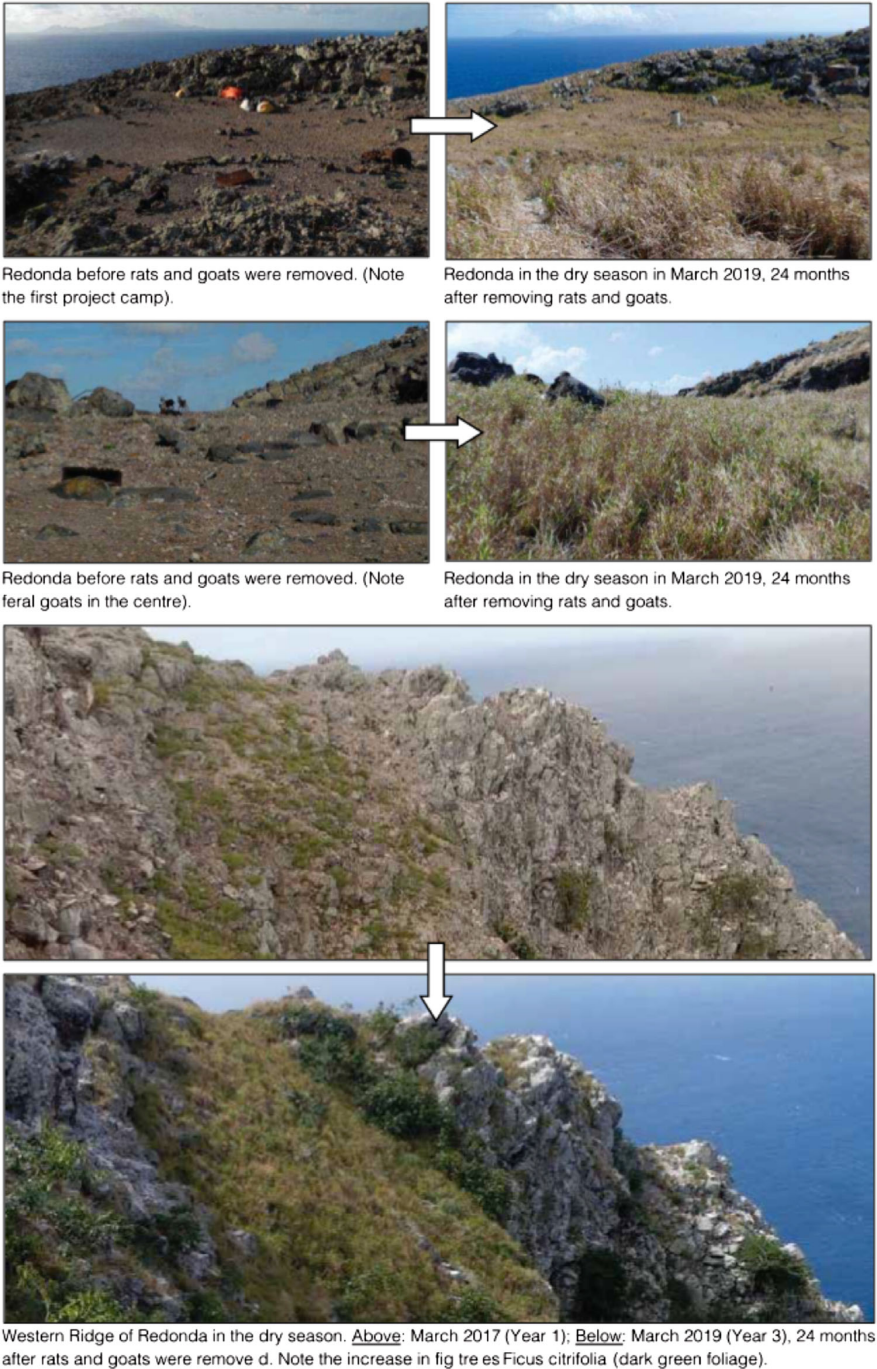
Pictures of Redonda from before and after the rat eradication and goat removal.

Following the removal of goats and rats in 2017, we saw a substantial increase in the relative density of *P. atratus* on Redonda. Both our transect and point count surveys told the same story of a rapid increase in population size. Granted, censuses based on visual observation can be biased by, for example, weather conditions or observer error. Despite the possible error intrinsic to visual estimates, we are confident that the number of lizards on the island has substantially increased since the invasive mammals were removed. This finding is consistent with studies of other species that have recorded rapid population growth in reptiles after removing invasive rats from islands, including *P. plei* on Dog Island, Anguilla, which increased by sixfold within 4 years of eradicating invasive *R. rattus* (Daltry & Bell 2018), while comparative studies in Antigua found rat‐free islands support a 3 times higher density of *P. griswoldi* than islands with *R. rattus* (Daltry *et al*. 2012; Lawrence *et al*. 2013). We suggest that the sharp increase in the *P. atratus* population on Redonda can be explained both by reduced direct predation by rats and the very rapid increase in vegetation and invertebrate populations on the island after the rats and goats were removed (Fig. 4). Analysis of fixed point photographs show a more than 20‐fold increase in plant biomass on Redonda between March 2017 and March 2019, while monitoring using standardized pitfall traps during the same period revealed an 8.6‐fold increase in the abundance of terrestrial invertebrates—an important food source for the lizards (JD and SC, unpublished data).

Additionally, the removal of these invasive rats and goats appears to have changed the lizards’ behavior, with the animals now allowing us to approach more closely before they flee. This behavioral shift suggests that the decrease in predation pressure has directly or indirectly rendered the lizards less wary, as is typically observed on islands (Cooper *et al*. 2014; Brock *et al*. 2014). Interestingly, flight distance did increase in 2018, suggesting that the lizards wait longer to run, but ultimately run farther, when confronted with a threat. However, the final distance from the observer still decreased, despite the greater flight distance, suggesting a lack of directionality in the escape path possibly due to the absence of predators. In addition to the effect of the absence of rats as potential predators, the differences in flight behavior may also have their origin in changes in habitat structure. Indeed, variation in habitat structure has been shown to affect escape tactics (Cooper & Whiting 2007). Thus, the increase in vegetation on the island following the removal of goats and rats may in itself have been a driver of variation in behavior. By the nature of the experiment, we cannot rule out the possibility that the lizards had become less wary of humans due to the presence of the teams removing the rats and goats. That said, because the team was small (fewer than 14 people at any time), we feel this is an unlikely explanation for the observed effect.

In addition to changes in lizard numbers and behavior, we also observed changes in morphology. More specifically, for their size, males had more robust heads in 2018, following the removal of rats and goats. Females, in contrast, showed no differences in head morphology when controlling for differences in snout–vent length between the years. The larger and more robust heads of males make sense as lizard numbers have dramatically increased. Males of many lizard species are known to be territorial and to actively defend their territories by biting (Husak *et al*. 2006a,b; Lailvaux & Irschick 2007). Most teiids are considered non‐territorial (Censky 1997), although males have been recorded following females and actively guarding them during the mating season (Ribeiro *et al*. 2011). We have observed *P. atratus* individuals fighting on numerous occasions during our surveys and so the increasing number of individuals on the island will likely result in more frequent and violent interactions between males, thus imposing selective pressures on head size (Husak *et al*. 2009). Whether the changes in head size that we observed just a short period of time after the eradication are due to differential survival of large‐headed males or the result of phenotypic plasticity remains to be tested.

Beyond the changes in head dimensions we also observed changes in limb dimensions, with males having longer fore‐ and hind limbs in 2018. Females, on the other hand only showed an increase in forelimb length, specifically the length of the two proximal limb segments, the humerus and radius. The longer limbs of males may, similarly to the increase in head dimensions, be related to increased numbers of interactions between males in relation to territory defense. Long limbs are directly related to sprint speed in lizards (Bauwens *et al*. 1995; Bonine & Garland 1999; Lowie *et al*. 2019) and have been shown to provide a fitness advantage in other species (Husak *et al*. 2006b). As such, males with longer limbs are likely to be faster runners and may be better at guarding females (Husak *et al*. 2008). However, females also showed differences in limb dimensions despite not being territorial. This suggests that other drivers of limb length may be responsible for the increase in forelimb length in both sexes. One possibility is the increase in vegetation observed on the island. As vegetation density and height increases, lizards will have to negotiate a more complex habitat structure that places novel demands on maneuverability. Previous studies on lizards have suggested that longer forelimbs resulting in more equal fore‐ and hind limb lengths may promote maneuverability (Vanhooydonck *et al*. 2001; Donihue 2016; Sathe & Husak 2018). Moreover, longer forelimbs may allow lizards to elevate the head higher, thereby providing greater visual range in the denser vegetation in 2018. As for the head dimensions, we cannot yet tell whether the observed changes are the result of differential survival of animals with different morphologies or whether these changes represent a phenotypically plastic response. However, irrespective of the underlying mechanism, our results show rapid changes in lizard density, behavior, and morphology following the eradication and removal of invasive mammals. Future follow‐up studies are needed to document the phenotypic trajectories of the population over medium‐ to long‐term time spans.

In conclusion, our data show how populations of native *Pholidoscelis* lizards can rebound rapidly after the removal of invasive alien rats and goats. Our data further show rapid changes in behavior and morphology resulting in changes in the phenotypic trajectory of the population, possibly en route toward the original phenotype that used to be present on the island of Redonda. These data show that targeted removals of invasive alien species can have rapid and important consequences for the behavior and morphology of island endemic species.

## CONFLICT OF INTEREST

The authors declare no conflicts of interest.

## References

[inz212500-bib-0001] Bauwens D , Garland T Jr , Castilla A , Van Damme R (1995). Evolution of sprint speed in lacertid lizards: morphological, physiological and behavioral covariation. Evolution; Internation Journal of Organic Evolution 49, 848–63. 10.1111/j.1558-5646.1995.tb02321.x 28564867

[inz212500-bib-0002] Bell EA , Daltry JC (2012). Feasibility study for the eradication of black rats *Rattus rattus* from Redonda, with new observations on the island's biodiversity and ecology. Report from Wildlife Management International Ltd and Fauna & Flora International to the Offshore Islands Conservation Programme, St John's, Antigua and Barbuda.

[inz212500-bib-1001] Bibby CJ , Burgess ND , Hill DA , Mustoe S (2000). Bird census techniques. Elsevier.

[inz212500-bib-0003] Bonine KE , Garland T Jr (1999). Sprint performance of phrynosomatid lizards, measured on a high‐speed treadmill, correlates with hindlimb length. Journal of Zoology 248, 255–65

[inz212500-bib-0004] Brock KM , Bednekoff PA , Pafilis P , Foufopoulos J (2014). Evolution of antipredator behavior in an island lizard species, *Podarcis erhardii* (Reptilia: Lacertidae): the sum of all fears? Evolution: An International Journal of Organic Evolution 69, 216–31. 10.1111/evo.12555 25346210

[inz212500-bib-0005] Breuil M , Schikorski D , Vuillaume B *et al*. (2020). Painted black: *Iguana melanoderma* (Reptilia, Squamata, Iguanidae) a new melanistic endemic species from Saba and Montserrat islands (Lesser Antilles). ZooKeys 926, 95–131. 32336922 10.3897/zookeys.926.48679 PMC7170970

[inz212500-bib-0006] Campbell K , Donlan CJ (2005). Feral goat eradications on islands. Conservation Biology 19, 1362–74.

[inz212500-bib-0007] Censky EJ (1997). Female mate choice in the non‐territorial lizard *Ameiva plei* (Teiidae). Behavioral Ecology and Sociobiology 40, 221–5.

[inz212500-bib-0008] Chynoweth MW , Litton CM , Lepczyk CA , Hess SC , Cordell S (2013). Biology and impacts of Pacific island invasive species. 9. *Capra hircus*, the feral goat (Mammalia: Bovidae). Pacific Science 67, 141–56.

[inz212500-bib-0009] Coblentz BE (1978). The effect of feral goats (*Capra hircus*) on island ecosystems. Biological Conservation 13, 279–86.

[inz212500-bib-0010] Colvocoresses J , Acosta A (2007). A large‐scale field comparison of strip transect and stationary point count methods for conducting length‐based underwater visual surveys of reef fish populations. Fisheries Research 85, 130–41.

[inz212500-bib-0011] Cooper WE Jr , Whiting M (2007). Universal optimization of flight initiation distance and habitat‐driven variation in escape tactics in a Namibian lizard assemblage. Ethology 113, 661–72.

[inz212500-bib-0012] Cooper WE Jr , Pyron RA , Garland T (2014). Island tameness: living on islands reduces flight initiation distance. Proceedings of the Royal Society B 281, 0133019. 10.1098/rspb.2013.3019 PMC3896029 24403345

[inz212500-bib-0013] Daltry JC (2006). Control of the black rat *Rattus rattus* for the conservation of the Antiguan racer *Alsophis antiguae* on Great Bird Island, Antigua. Conservation Evidence 3, 28–9.

[inz212500-bib-0014] Daltry JC (2007). An introduction to the herpetofauna of Antigua, Barbuda and Redonda, with some conservation recommendations. Applied Herpetology 4, 97–130.

[inz212500-bib-0015] Daltry JC , Bell EA (2018). Can brodifacoum save endangered species? Recent experiences from the West Indies. Outlooks on Pest Management 18, 80–5.

[inz212500-bib-0016] Daltry JC , James KJ , Otto A , Ross TN (2012). Evidence that eradicating black rats has boosted the recovery of rare reptiles and seabirds on Antiguan islands. In: Vernier JL , Burac M , eds. Biodiversité Insulaire: La Flore, la Faune et l'Homme dans les Petites Antilles. Université des Antilles et de la Guyane, pp. 141–5.

[inz212500-bib-0017] Donihue CM (2016). Microgeographic variation in locomotor traits among lizards in a human‐built environment. PeerJ 4, e1776; 10.7717/peerj.1776. 26989616 PMC4793326

[inz212500-bib-0018] Donihue CM , Giller G , Herrel A (2017). *Pholidoscelis atrata* (Redonda ground lizard) diet. Herpetological Review 48, 655.

[inz212500-bib-0019] Donihue CM , Herrel A , Fabre A‐C *et al*. (2018). Hurricane‐induced selection on the morphology of an island lizard. Nature 560, 88–92. 30046104 10.1038/s41586-018-0352-3

[inz212500-bib-0020] Duron Q , Bourguet E , De Meringo H , Million A , Vidal E (2017). Invasive rats strengthen predation pressure on bird eggs in a South Pacific island rain forest. Current Zoology 63, 583–90. 29492018 10.1093/cz/zox009 PMC5804218

[inz212500-bib-0021] Goicoechea N , Frost DR , De la Riva I *et al*. (2016). Molecular systematics of teioid lizards (Teioidea/Gymnophthalmoidea: Squamata) based on the analysis of 48 loci under tree‐alignment and similarity‐alignment. Cladistics 32, 624–71. 34727678 10.1111/cla.12150

[inz212500-bib-0022] Harper GA , Bunbury N (2015). Invasive rats on tropical islands: Their population biology and impacts on native species. Global Ecology and Conservation 3, 607–27.

[inz212500-bib-0023] Havery S , Oppel S , Cole N , Duffield N (2018). Density of three skink species on a sub‐tropical pacific island estimated with hierarchical distance sampling. Herpetological Conservation and Biology 13, 507–16.

[inz212500-bib-0024] Hedges SB , Conn CE (2012). A new skink fauna from Caribbean islands (Squamata, Mabuyidae, Mabuyinae). Zootaxa 3288, 1–244.

[inz212500-bib-0025] Herrel A , Huyghe K , Vanhooydonck B *et al*. (2008). Rapid large‐scale evolutionary divergence in morphology and performance associated with exploitation of a different dietary resource. PNAS 105, 4792–5. 18344323 10.1073/pnas.0711998105 PMC2290806

[inz212500-bib-0026] Husak JF , Lappin AK , Fox SF , Lemos‐Espinal JA (2006a). Bite‐force performance predicts dominance in male venerable collared lizards (*Crotaphytus antiquus*). Copeia 2006, 301–6.

[inz212500-bib-0027] Husak JF , Fox SF , Lovern MB , Van Den Bussche RA (2006b). Faster lizards sire more offspring: sexual selection on whole‐animal performance. Evolution: An International Journal of Organic Evolution 60, 2122–30. 17133868

[inz212500-bib-0028] Husak JF , Fox SF , Van Den Bussche RA (2008). Faster male lizards are better defenders not sneakers. Animal Behaviour 75, 1725–30.

[inz212500-bib-0029] Husak JF , Lappin AK , Van Den Bussche RA (2009). The fitness advantage of a high performance weapon. Biological Journal of the Linnean Society 96, 840–5.

[inz212500-bib-0030] Lailvaux SP , Irschick DJ (2007). The evolution of performance‐based male fighting ability in Caribbean *Anolis* lizards. American Naturalist 170, 573–86. 10.1086/521234 17891736

[inz212500-bib-0031] Lawrence N , James K , Otto A , Ross TN , Daltry JC (2013). Is eradicating rats worth it? Impacts observed on Antigua's offshore islands. Program and Abstracts: Society for the Conservation and Study of Caribbean Birds, 19th Regional Meeting, 26–31 Jul 2013, Georgetown, Grenada.

[inz212500-bib-0032] Lawrence SN , Challenger S , Bell EA , Daltry JC , Steele SM (2017). Relocating feral goats and eradicating Eurasian ship rats to save Redonda's birds. Program and Abstracts: BirdsCaribbean, 21st Regional Meeting, Cuba.

[inz212500-bib-0033] Lazell JD (1972). The Anoles (Sauria, Iguanidae) of the Lesser Antilles (Bulletin of the Museum of Comparative Zoology), Vol. 143. Harvard University, Cambridge, MA.

[inz212500-bib-0034] Lowie A , Gillet E , Vanhooydonck B , Irschick DJ , Losos JB , Herrel A (2019). Do the relationships between hind limb anatomy and sprint speed variation differ between sexes in *Anolis* lizards? Journal of Experimental Biology 222, 188805. 10.1242/jeb.188805 30683664

[inz212500-bib-0035] Meyer J‐Y , Butaud J‐F (2008). The impacts of rats on the endangered native flora of French Polynesia (Pacific Islands): drivers of plant extinction or coup de grâce species? Biological Invasions 11, 1569–85.

[inz212500-bib-0036] Pascal M , Brithmer R , Lorvelec O , Vénumière N (2004). Conséquences sur l'avifaune nicheuse de la réserve naturelle de l’îlets de Sainte‐Anne (Martinique) de la récente invasion du rat noir (*Rattus rattus*) établies à l'issue d'une tentative d’éradication. Revue d’Écologie, Terre Vie 59, 309–18.

[inz212500-bib-0037] Pascal M , Lorvelec O , Bretagnolle V , Culioli J (2008). Improving the breeding success of a colonial seabird: a cost‐benefit comparison of the eradication and control of its rat predator. Endangered Species Research 4, 267–76.

[inz212500-bib-0038] Parmesan C (2006). Ecological and evolutionary responses to recent climate change. The Annual Review of Ecology, Evolution, and Systematics 37, 637–69.

[inz212500-bib-0039] Pender RJ , Shiels AB , Bialic‐Murphy L , Mosher SM (2013). Large‐scale rodent control reduces pre‐ and post‐dispersal seed predation of the endangered Hawaiian lobeliad, *Cyanea superba* subsp. *superba* (Campanulaceae). Biological Invasions 15, 213–23.

[inz212500-bib-0040] Ribeiro LB , Gogliath M , Fernandes R , de Sales D , Freire EMX (2011). Mating behavior and female accompaniment in the whiptail lizard *Cnemidophorus ocellifer* (Squamata, Teiidae) in the Caatinga region of northeastern Brazil. Biota Neotropica 11, 4.

[inz212500-bib-0041] Russell JC , Towns DR , Clout M (2008). Review of Rat Invasion Biology: Implications for Island Biosecurity (Science for Conservation), Vol. 286. Department of Conservation, Wellington, New Zealand.

[inz212500-bib-0042] Russell JC , Meyer J‐Y , Holmes ND , Pagad S (2017). Invasive alien species on islands: impacts, distribution, interactions and management. Environmental Conservation 44, 359–70.

[inz212500-bib-0043] Sathe EA , Husak JF (2018). Substrate‐specific locomotor performance is associated with habitat use in six‐lined racerunners (*Aspidoscelis sexlineata*). Biological Journal of the Linnean Society 124, 165–73.

[inz212500-bib-0044] Simberloff D , Martin J‐L , Genovesi P *et al*. (2013). Impacts of biological invasions: What's what and the way forward. Trends in Ecology & Evolution 28, 58–66. 22889499 10.1016/j.tree.2012.07.013

[inz212500-bib-0045] Sparrow HR , Sisk TD , Ehrlich PR , Murphy DD (1994). Techniques and guidelines for monitoring neotropical butterflies. Conservation Biology 8, 800–9.

[inz212500-bib-0046] Spatz DR , Zilliacus KM , Holmes ND *et al*. (2017). Globally threatened vertebrates on islands with invasive species. Science Advance 3, e1603080. 10.1126/sciadv.1603080 PMC5656423 29075662

[inz212500-bib-0047] St Clair JJH (2011). The impacts of invasive rodents on island invertebrates. Biological Conservation 144, 68–81.

[inz212500-bib-0048] Towns DR , Atkinson IAE , Daugherty CH (2006). Have the harmful effects of introduced rats on island been exaggerated? Biological Invasions 8, 863–91.

[inz212500-bib-0049] Vanhooydonck B , Van Damme R , Aerts P (2001). Ecomorphological correlates of habitat partitioning in Corsican lacertid lizards. Functional Ecology 14, 358–68.

[inz212500-bib-0050] Ward A , Daltry JC , Bunting R , Ridley R (2010). An assessment of the feasibility of removing goats from Dog Island, Anguilla, British West Indies, with additional observations on the island's ecology. Department of Environment, Food and Rural Affairs, York, and Fauna & Flora International, Cambridge, UK.

